# Extraction, purification, structural characterization, and antioxidant activity of a novel polysaccharide from *Lonicera japonica* Thunb.

**DOI:** 10.3389/fnut.2022.1035760

**Published:** 2022-11-01

**Authors:** Feiyu An, Guangyu Ren, Junrui Wu, Kaixin Cao, Mo Li, Yumeng Liu, Yanfeng Liu, Xinyu Hu, Meijun Song, Rina Wu

**Affiliations:** ^1^College of Food Science, Shenyang Agricultural University, Shenyang, China; ^2^Key Laboratory of Microbial Fermentation Technology Innovation, Shenyang, China; ^3^Engineering Research Center of Food Fermentation Technology, Liaoning, China

**Keywords:** honeysuckle, polysaccharide, antioxidant activity, oxidative stress, structural characterization

## Abstract

A novel water-soluble polysaccharide (HEP-4) with a molecular weight of 1.98 × 10^5^Da was extracted from honeysuckle. Structural characterization was performed using high-performance liquid chromatography (HPLC), gas chromatography, Fourier transform-infrared (FT-IR) spectrum, nucleus magnetic resonance (NMR) spectra, and scanning electron microscopy. The results showed that HEP-4 is primarily composed of mannose, rhamnose, galacturonic acid, glucose, galactose, and arabinose with a mole ratio of 6.74:1.56:1.04:14.21:4.31:5.4, and the major types of the glycosidic bond types of HEP-4 were 1-α-D-Glc*p*, 1,4-β-D-Glc*p*, 1-β-D-Ara*p*, 1,3,4-β-D-Ara*p*, and 1,3,6-β-D-Man*p*. The results of bioactivity experiments revealed that HEP-4 had antioxidant *in vitro*. In addition, HEP-4 inhibited H_2_O_2_-induced oxidative damage and increased the activity of HepG2 cells by reducing MDA levels and inhibiting ROS production. Meanwhile, HEP-4 significantly enhanced the activities of GSH-Px and CAT, indicating that HEP-4 exerts a protective effect on H_2_O_2_-induced oxidative stress. These results indicate that HEP-4 could be a potential natural antioxidant.

## Introduction

Honeysuckle (*Lonicera japonica* Thunb.) belongs to the *Caprifoliaceae* family of medicinal plants (1). It is widely distributed in the Yellow River and Yangtze River basins of China and is largely planted in Hunan, Henan, Shandong, and other provinces. Several studies have reported that Honeysuckle has anti-cancer, anti-inflammatory, anti-allergic, antipyretic, and other effects (2–5), which could be related to the various bioactive components present in Honeysuckle, such as polysaccharides, saponins, tannins, and flavonoids, etc., (6, 7).

Of the numerous functional components in honeysuckle, polysaccharides are especially important as a pleiotropic biological response modifier, and their pharmacological activities largely include antioxidant (5), anti-allergy (6), immune regulation (8), and neuroprotection (9), etc. Among these, the antioxidant capacity has received extensive attention recently, and several researchers have studied the antioxidant activities of other natural plant polysaccharides such as areca nut (10), jujube (11), Tossa jute leaves (12), and wheat bran (13). Relevant research results demonstrate that plant polysaccharides exert a strong antioxidant activity and low side effects, and can be used as an important source of antioxidants to prevent the risk of free radical injury and the production of several related diseases (14).

Several researchers believe that the monosaccharide composition, molecular weight, functional groups, glycosidic bond composition, branching, and conformation of polysaccharides are closely related to their antioxidants and other biological activities (15). For instance, Zhang et al. reported that pinecone polysaccharides with a high content of L-rhamnose and L-arabinose had better antioxidant activity (16). Zhi et al. found that water-soluble polysaccharides with antioxidant activity isolated from roots of *Dioscorea opposita* Thunb. may be related to its low molecular weight (17). Therefore, to reveal and explore the structural-biological activity relationship of polysaccharides, we need to conduct structure-biological activity studies on various polysaccharides from different sources and conduct comparative analysis.

At present, the major antioxidant active polysaccharide components in the total sugar of honeysuckle are not known, hindering the in-depth study of the structure and antioxidant activity of honeysuckle polysaccharides. In this study, we extracted a novel polysaccharide (HEP-4) from honeysuckle and characterized its structural features. In addition, the antioxidant activity of HEP-4 was evaluated, and the protective effect of HEP-4 on H_2_O_2_-induced oxidative stress was studied in combination with cell experiments. The research results provide a basis for the comprehensive development and utilization of honeysuckle polysaccharides.

### Materials and methods

### Materials

The honeysuckle was purchased from the Shenyang of Liaoning province, China. DEAE-52 cellulose, Sephadex G-75, Vitamin-C, and monosaccharide standards were purchased from Yuanye Bio-Technology Co., Ltd. (Shanghai China). DMEM cell culture, fetal bovine serum (FBS), and penicillin were purchased from HyClone (Logan, Utah, USA). HepG2 cell line was purchased from Stem Cell Bank, Chinese Academy of Sciences.

### Extraction, isolation, and purification of polysaccharides

The peel of honeysuckle was removed and it was lyophilized to constant weight and ground. The powder was immersed in 95% ethanol to remove the lipids/pigments and filtered (18). The prepared honeysuckle powder (100 g) was extracted with distilled water (1,000 ml) at 80°C for 3 h. All extraction solutions were collected and concentrated using the vacuum rotary evaporator (60°C), to which 95% ethanol was added and left to stand for 24 h; the solution was centrifuged (10,000 rpm, 10 min) and the precipitate was dissolved in ultrapure water (19).

Proteins were removed using the Sevag method as described previously (20). Next, the crude polysaccharide sample obtained after elution was equipped with a DEAE-52 cellulose column (2.6 cm × 30 cm), which was stepwise eluted with distilled water and 0.1, 0.2, 0.3, 0.4, and 0.5 M gradients of NaCl solutions sequentially at a flow rate of 1.5 ml/min. All tubes were quantified by phenol–sulfuric acid method. Next, the Sephadex G-75 gel chromatography column (2.6 cm × 100 cm) was used for further purification. Thus, five fine polysaccharide components HEPs were obtained. After comprehensive comparison of the antioxidant activities of the scavenging effects of ABTS⋅^+^, DPPH⋅, superoxide, and hydroxyl, HEP-4 was selected and freeze-dried to obtain purified polysaccharide powder for further use.

### Evaluation of structure characteristics of HEP-4

#### Chemical analysis

The phenol-sulfuric acid method, Coomassie brilliant blue method, and m-hydroxy biphenyl method were used to evaluate the content of the overall sugar, proteins, and uronic acid in the polysaccharide fraction.

#### Determination of molecular weight distribution

The mean molecular weight of the sample was determined by high-performance gel permeation chromatography (21). The column was eluted with 0.2 M Na_2_SO_4_ solution at a flow rate of 0.6 ml/min. The column temperature was maintained at 35 ± 0.1°C. The injection volume was 20 μL and the average molecular weight of HEP-4 was calculated by the standard curve, which was established using the T-series dextran.

#### Monosaccharide composition analysis

The monosaccharide composition of HEP-4 was analyzed according to a previously described method with certain modifications (22) and the detailed method is presented in Supplementary method 1.

#### Methylation analysis

Methylation analysis of polysaccharides was performed as described previously with minor modifications (23), and the specific experimental method is presented in Supplementary method 2.

#### Periodate oxidation and Smith degradation reaction

Periodate oxidation and Smith degradation of HEP-4 were conducted according to the method described by Meng et al. (24). The standard curve equation of sodium periodate solution was obtained as follows Y = 2.222X + 3.6491, R2 = 0.9991.

#### Fourier transform-infrared spectroscopy analysis

The infrared spectrum of HEP-4 was determined using an FT-IR spectrometer (Nicolet 6,700, Thermo Fisher Scientific, Waltham, MA, USA). The dried HEP (2 mg) was ground with KBr (200 mg) and pressed into pellets for FT-IR measurement in the frequency range of 4,000–400 cm^–1^ at a resolution of 4 cm^–1^ (25).

#### Nucleus magnetic resonance analysis

HEP-4 (60 mg/ml) was dissolved and deutero-exchanged with D_2_O and afterward re-dissolved in 0.5 ml of D_2_O. The ^1^H NMR and ^13^C NMR of the polysaccharides were recorded with a VNMRS600 NMR spectrometer (Agilent, CA, USA) at 60°C. The MestReNova software was used to process the NMR data, and chemical shifts (δ) are expressed in ppm (26).

#### Scanning electron microscopy analysis

The morphology of HEP-4 was evaluated by scanning electron microscopy. An appropriate amount of HEP-4 sample was fixed and coated with a layer of conductive gold film and afterward examined using an SEM system (SU1510, Hitachi, Tokyo, Japan) (27).

### Antioxidant activity

The DPPH⋅scavenging activity of HEP-4 was assessed according to the reported method with minor modifications (28), and the specific experimental methods were presented in Supplementary method 3. The superoxide anion scavenging activities were determined using the method described by Liang et al. (29) and the specific experimental method is presented in Supplementary method 4. The detailed method of the ABTS⋅^+^ radical scavenging assay is presented in Supplementary method 5. The hydroxyl radical scavenging activity of HEP-4 was determined as described previously in the literature (30) and the detailed method is presented in Supplementary method 6.

### Inducing HepG2 cells with H_2_O_2_

#### Cell culture and treatments

HepG2 cells were cultured in DMEM with FBS (10% v/v) and penicillin (1% v/v) in 95% air and 5% CO_2_ humidified atmosphere at 37°C. After passaging thrice, the cells were used to seed 6-well culture plates.

HepG2 cells were treated with different concentrations of HEP-4 (200, 400, and 800 μg/mL) for 4 h and washed with warm PBS thrice. In addition, 800 μg/mL Vc was used as the control group. Except for the blank group, the cells of other groups were added to 6 mmol/L H_2_O_2_ for 1.5 h and washed with PBS. Next, 200 μL of the cracking solution was added to each well and placed in a refrigerator at 4°C for 15 min. Afterward, it was sucked out and collected in 1.5 mL centrifuge tubes.

#### Hoechst 33342 fluorescence staining

HepG2 cells were grown in five glass-bottom cell culture dishes for 24 h and treated with 6 mmol/L H_2_O_2_ and different concentrations (200, 400, and 800 μg/mL) of HEP-4. The cells were washed twice with PBS. Next, the cells were stained with 500 μL of the medium and mixed with 5 μL of Hoechst 33342 for 10–20 min at 25°C. Finally, the washed cells were observed using a laser confocal microscope (31).

#### Analysis of cell reactive oxygen species

To visualize and quantify intracellular reactive oxygen species (ROS) production, the generation of ROS was monitored using a fluorometric intracellular ROS kit (Nanjing Jiancheng Bio Co., Ltd., Nanjing, Jiangsu, China).

#### Analysis of glutathione peroxidase catalase, malondialdehyde, and catalase

To study the influence of HEP-4 samples on the oxidative stress status of H_2_O_2_-induced HepG2 cells, the content of protein was measured using a bicinchoninic acid kit (Beyotime, China). The activities of glutathione peroxidase catalase (GSH-Px) (Nanjing Jiancheng, China) and catalase (CAT) (Solarbio, Beijing, Hebei, China), and the content of malondialdehyde (MDA) (Nanjing Jiancheng, China) were determined according to the manufacturer’s instructions.

### Statistical analysis

The experiments were repeated thrice. All data are expressed as mean ± standard deviation (SD). Statistical significance was calculated by one-way analysis of variance (ANOVA), and the difference was considered significant at *P* < 0.05.

## Results and discussion

### Purification of HEP-4 and determination of molecular weight

**FIGURE 1:**
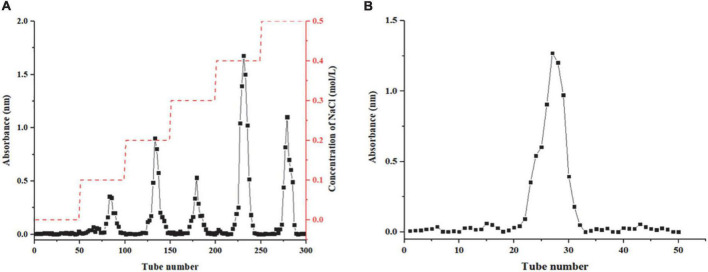
Results column chromatography results of honeysuckle polysaccharide. **(A)** DEAE-52 column chromatography of honeysuckle polysaccharide. **(B)** Sephadex G-75 column chromatography of HEP-4.

In this study, *Honeysuckle* as raw material, crude polysaccharides were extracted by hot water extraction method, and the protein was removed by the Sevag method. The polysaccharide content in crude HEP was 2.09 ± 0.21%. Five fractions (HEP-1, HEP-2, HEP-3, HEP-4, and HEP-5) were separated on the DEAE-52 column, among which HEP-4 had the highest elution peak (Figure 1A) and had the highest resistance to oxidative activity (data not shown). Therefore, we further purified HEP-4 crude fractions using gel filtration chromatography (Figure 1B), and afterward collected and lyophilized them to obtain HEP-4. The basic chemical composition analysis of the lyophilized fraction revealed that the contents of total sugar, proteins, and uronic acid in HEP-4 were 88.62 ± 2.04%, 1.71 ± 0.58%, and 2.20 ± 0.09%, respectively. In addition, the average molecular weight of HEP-4 was estimated to be 1.98 × 10^5^ Da. Zhang et al. reported that the average molecular weights of four honeysuckle water-soluble polysaccharides ranged from 1.9 × 10^4^ to 3.84 × 10^5^ Da, among which the average molecular weights of antioxidant polysaccharides were relatively high and similar to HEP-4 (5). Sheng et al. proposed that polysaccharides from the fern *Polydonta* had strong hydroxyl radical scavenging ability at low molecular weight, whereas a strong reducing ability and DPPH⋅ radical scavenging ability at the high molecular weight (32). Therefore, we believe that the effect of molecular weight of polysaccharides on their antioxidant activity is not absolute, and the joint effects of other structural factors need to be studied.

### Monosaccharide composition of HEP-4

As shown in Supplementary figure 1. HEP-4 is primarily composed of mannose, rhamnose, galacturonic acid, glucose, galactose, and arabinose in a ratio of 6.74:1.56:1.04:14.21:4.31:5.4. Compared with the results of a previous study on honeysuckle polysaccharides (5, 9), we found differences in the monosaccharide composition, which could be attributed to the differences in separation and purification methods and raw materials, which will also lead to differences in their active functions.

It has been demonstrated that monosaccharide composition is intricately related to the antioxidant activity of polysaccharides. For example, Mehmood et al. reported that *Tricholoma lobayense* heteroglycan with high glucose and galactose content showed a strong antioxidant activity (33). In addition, the *T. lobayense* heteroglycan contains a certain amount of rhamnose, which was the reason for its high antioxidant activity. In this study, HEP-4 showed a high content of glucose and mannose, and rhamnose, indicating its high potential for antioxidant activity.

### Structural characterization of HEP-4

#### Methylation analysis of HEP-4

In addition to the composition of monosaccharides, the activity of polysaccharides is intricately related to the changes in chemical structures such as spatial conformation and the type of glycoside linkage (10). Methylation analysis is widely used to identify the linkage types between glucosyl residues. The analysis result is summarized in Table 1. Three types of Glc residues are mainly composed of 2,3,6-Me_3_-Glc*p*, 2,3,4,6-Me_4_-Glc*p*, and 2,4,6-Me_3_-Glc*p* with a molar ratio of 50.8:24.5:16.59. In addition, two types of Ara and one type of Man were detected, which were characterized as 2,3,5-Me_3_-Ara*f*, 2,3,-Me_2_-Ara*f*, and 2,4-Me_2_-Man*p*. The results showed that HEP-4 was primarily induced by 1-linked-Glc, 1,4-linked-Glc, 1,3-linked-Glc, 1-linked-Ara, 1,3,4-linked-Ara, and 1,3,6-linked-Man, and the highest content of 1,4-linked-Glc was probably contained in the main chain. The results of the analysis were consistent with those of monosaccharide composition analysis.

**TABLE 1:** GC-MS results of methylation analysis of HEP-4.

Peaks	T_R_ (min.)	Methylated sugar	Molar ratio (%)	Linkage type	Major mass fragment (m/z)
1	10.017	2,3,5-Me_3_-Ara*f*	3.77	Ara*f*-(1→	43,71,87,101,117,129,145,161
2	16.421	2,4-Me_2_-Man*p*	5.22	→3,6)-Man*p*-(1→	43,71,87,99,101,117,129,159,161,189
3	16.815	2,3,-Me_2_-Ara*f*	11.92	→3,4)-Ara*f*-(1→	43,71,85,99,101,117,127,159,161
4	20.460	2,3,4,6-Me_4_-Glc*p*	24.5	Glc*p*-(1→	43,71,87,101,117,129,145,161,205
5	22.757	2,3,6-Me_3_-Glc*p*	30.8	→4)-Glc*p*-(1→	43,71,87,99,101,113,117,129,131,161,173
6	26.533	2,4,6-Me_3_-Glc*p*	16.59	→3)-Glc*p*-(1→	43,45,71,87,101,117,129,159,161,234

#### Sodium periodate oxidation and Smith degradation reaction

The sodium periodate oxidation results showed that 1 M glycosyl consumed 0.7491 mol of NaIO4 and liberated 0.007 mol of formic acid. The consumption of NaIO4 was more than double that of HCOOH, indicating the possible existence of 1→2, 1→2,6, 1→4, or 1→4,6, as well as 1→ or 1→6 linked glycosidic bonds (34). Furthermore, the GC analysis of the products of Smith degradation showed that it contained glycerin, further indicating that monosaccharides of HEP-4 could be connected largely by 1→2 or 1→2,6, and 1→ or 1→6 linked glycosidic bonds (34). Additionally, the presence of galactose was indicative of HEP-4 with the existence of 1→3, 1→3,4, and 1→3,6, linkages that cannot be oxidized (24).

#### Fourier transform-infrared analysis

Supplementary figure 2 shows the results of infrared spectrum analysis of honeysuckle polysaccharide. We obtained a broad and strong absorption peak of O-H stretching vibration peak between 3,600 and 3,400 cm^–1^, which was the characteristic absorption peak of polysaccharides (35). The absorption peak at 2,925.71 cm^–1^ is the C-H stretching vibration peak (36). The absorption band generated at 1,608.44 cm^–1^ could be the C-O stretching vibration peak in the sample. The absorption peak observed at 1,385.08 cm^–1^ could be attributed to the variable-angle vibration of C-H (37). The two strong peaks observed at 1,272.83 cm^–1^ and 1,049.98 cm^–1^ indicated that the polysaccharide sample contained a pyran polysaccharide (38).

#### Nucleus magnetic resonance spectroscopy analysis of HEP-4

The NMR spectroscopy can be used to confirm monosaccharides, identify alpha or beta-anomeric configurations, and elucidate glycosidic bonds (39). In ^1^H NMR, the chemical shift δ is greater than 5.0 ppm, indicating the α configuration of the heterotopic hydrogen of the polysaccharide molecule, whereas a chemical shift δ of less than 5.0 ppm indicates the β configuration of the heterotopic hydrogen of the polysaccharide molecule. The ^1^H NMR spectrum of typical polysaccharides was mostly in the range of 3–5 ppm. The chemical shifts from 4.48 to 3.4 ppm in the 1H NMR spectrum were sugars H-2 to H-6 signal, and 1.20 ppm was assigned to the methyl group in the rhamnose residue. In addition, 3.2–4.0 ppm was the sugar ring proton signal (40). In ^13^C NMR, the C2-C6 resonance signals were located at the region of 80.99 of 59.86 ppm. The chemical shifts of 109.9 of 96.4 ppm corresponded to the signal of the terminal sugar. In addition, 96.4–96.6 ppm corresponded to the terminal carbon signal of glucose (41).

As shown in Supplementary figure 3A. The ^1^H NMR proton spectrum signals of HEP-4 were largely concentrated between 3.40 and 5.15 ppm. There was no proton signal at 5.40 ppm, indicating that HEP-4 was composed of glucopyranose. Furthermore, we observed only a weak signal at 5.14 ppm, which was presumed to be α-glucose signal. The chemical shifts of HEP-4 from 4.70 to 3.41 ppm in the ^1^H NMR spectrum corresponded to sugars H-2 to H-6 signals. In addition, the band at 1.175 ppm indicated that the structure of polysaccharides could contain methyl groups of rhamnose residues. As shown in Supplementary figure 3B, HEP-4 showed several anomeric carbon signals from 95.0 to 110.0 ppm and multiple anomeric carbon signals in a broad region from 84.0 to 60.9 ppm. The resonances at 68.46, 71.86, 72.44, 62.45, and 66.36 ppm were assigned to C-2, C-3, C-4, C-5, and C-6, respectively. In addition, the NMR analysis data were consistent with the other structural characterization results described above.

In summary, the backbone of HEP-4 was 1,4-β-D-Glc*p*, 1-α-D-Glc*p*, and other rhamnose residues branched at 1-β-D-Ara*p*, 1,3,4-β-D-Ara*p*, or 1,3,6-β-D-Man*p*. Compared with the previous studies on honeysuckle polysaccharides, the structural characteristics of the isolated polysaccharide in this study are different, and it is a novel polysaccharide that has not been reported.

#### Scanning electron microscopy analysis of HEP-4

**FIGURE 2:**
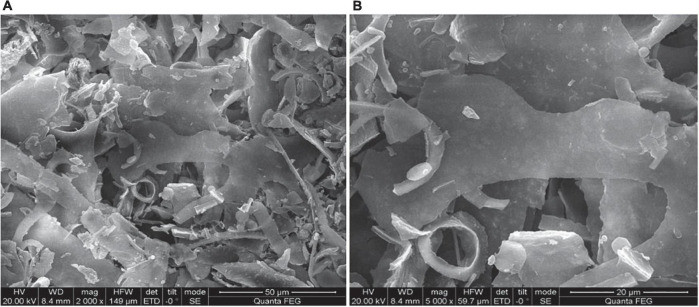
Scanning electron microscopy (SEM) images of HEP-4. **(A)** Magnification 2000× and **(B)** Magnification 5000×.

The surface morphology of HEP-4 was analyzed by SEM. As shown in Figure 2, HEP-4 exhibited a mixture of irregular structures at a magnification of 2,000× and 5,000×. The majority of them were flaky, and the others were heterogeneous granule shapes with no surface pores, similar to the microstructure of the blue honeysuckle polysaccharides studied by Ma et al. (42).

### Antioxidant activity of HEP-4

**FIGURE 3:**
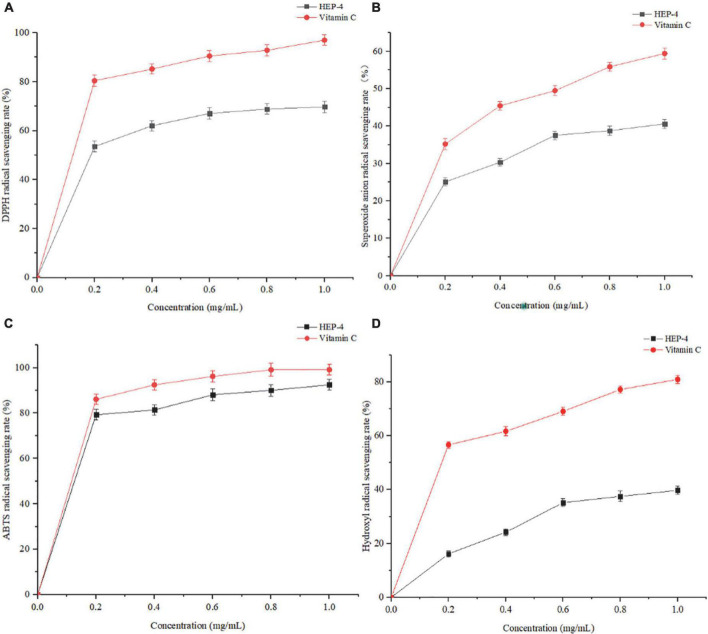
Antioxidant activity results of HEP-4. **(A)** DPPH⋅ radical scavenging assay, **(B)** Superoxide radical scavenging assay, **(C)** ABTS radical scavenging assay, **(D)** and hydroxyl radical scavenging assay.

DPPH⋅ free radical scavenging assay is widely used to evaluate the antioxidant activity *in vitro* (43). As shown in Figure 3A, the polysaccharide samples showed DPPH⋅ scavenging activity. With an increase in the concentrations of polysaccharides from 0 to 0.6 mg/mL, the DPPH⋅ radical scavenging activity increased, showing a positive linear correlation. At a concentration of 1.0 mg/mL, the scavenging rate of HEP-4 on DPPH⋅ free radical reached 69.73%, and the scavenging rate of Vc on DPPH⋅ free radical reached 97.01%. Therefore, we speculated that polysaccharides of honeysuckle had a certain role in scavenging DPPH⋅ free radical activity.

As shown in Figure 3B, with an increase in the HEP-4 concentration, the scavenging rate of superoxide free radical increased. The scavenging rate of HEP-4 on superoxide free radical reached 40.62%, and that of Vc reached 59.43%. Therefore, HEP-4 exerts a certain scavenging effect on superoxide free radicals; however, its scavenging ability is lower than that of Vc at the same concentration.

The ABTS⋅^+^ free radical scavenging assay is used to evaluate the total antioxidant capacity of natural products. The relevant test results showed that the positive control (Vc) presented a strong dose-dependent scavenging activity, and HEP-4 also established a good scavenging effect on ABTS⋅^+^ free radicals (Figure 3C). Within the range of mass concentration of 0–1.0 mg/mL, the scavenging ability of HEP-4 on ABTS⋅^+^ free radical was gradually enhanced. At a concentration of 1.0 mg/ml, the scavenging rate of HEP-4 on ABTS⋅^+^ free radical was 92.47%, and that of Vc on ABTS⋅^+^ free radical was 99.12%. These results indicated that honeysuckle polysaccharides should be explored as potential antioxidants.

Among the reactive oxygen species, hydroxyl radical is considered the most harmful free radical, which can stimulate the peroxidation of biomacromolecules in cells (44). As shown in Figure 3D, the scavenging rate of polysaccharides increased with an increase in their concentrations, followed by a flat trend. When the concentration was 1.0 mg/mL, the scavenging rate reached 39.78%, and the scavenging rate of Vc was up to 80.99%.

The above-mentioned four antioxidant capacity detection methods were used to systematically evaluate the antioxidant activity of honeysuckle polysaccharides (HEP-4) from different perspectives. These methods avoided the one-sidedness and inadequacy of evaluating the antioxidant activity of a single antioxidant method. In summary, antioxidant activity test results and comparison with previous research results of honeysuckle water-soluble polysaccharides (5) exhibited that HEP-4 has a strong scavenging activity against DPPH⋅, superoxide, ABTS⋅^+^, and hydroxyl radicals. The antioxidant activities of HEP-4 in different reaction systems vary, which could be related to certain structural features of polysaccharides. The results could lay the foundation for the research and comprehensive development and utilization of honeysuckle polysaccharides in natural antioxidants.

### Hoechst 33342 fluorescent staining analysis

**FIGURE 4:**
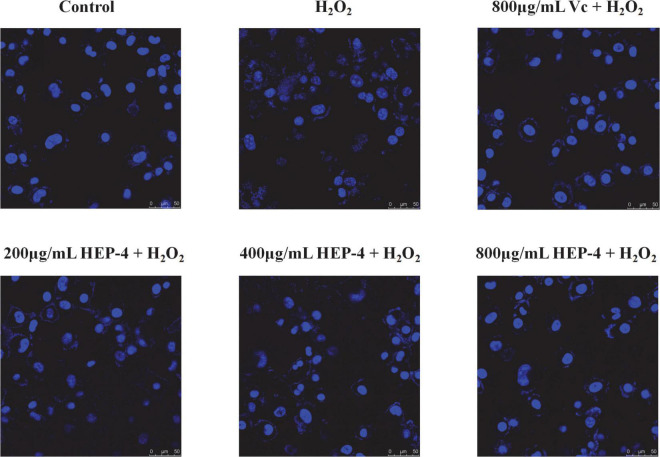
Nuclear condition after Hoechst 33258 staining.

Hoechst 33342 is a cell-permeable blue fluorescent dye (45) that stains nuclei with blue fluorescence. As shown in Figure 4, the effect of different concentrations of HEP-4 on H_2_O_2_-stimulated HepG2 cells was observed. In the control group, HepG2 cells have a uniform nuclear size with clear edges. After treatment with H_2_O_2_ alone, certain cells displayed apoptotic morphological changes such as blurred edges and increased internuclear spacing. In contrast, HEP-4-treated cells significantly reduced the degree of apoptosis in a concentration-dependent manner. The results showed that HEP-4 significantly inhibited the formation of apoptotic and relieved nuclear shrinkage induced by oxidative stress on HepG2 cells.

### Effects of HEP-4 on intracellular reactive oxygen species levels of HepG2 cells

**FIGURE 5:**
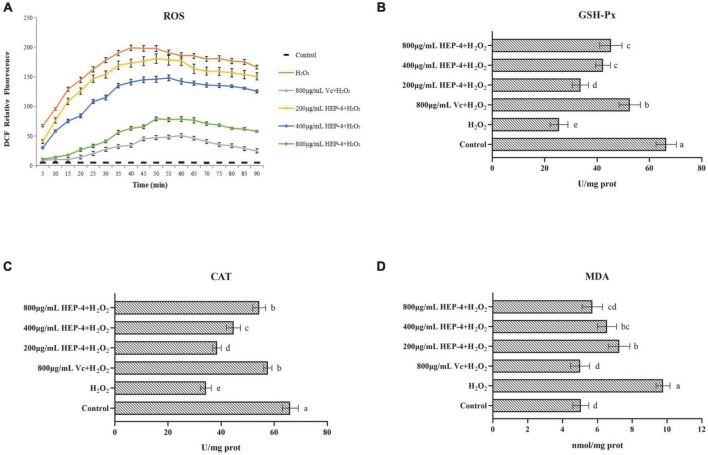
HEP-4 alleviates H_2_O_2_-induced oxidative stress. **(A)** Effect of HEP-4 on H_2_O_2_-induced ROS generation in HepG2 cells. **(B)** Effect of HEP-4 on glutathione peroxidase (GSH-Px). **(C)** Effect of HEP-4 on catalase (CAT). **(D)** Effect of HEP-4 on malondialdehyde (MDA) level. Values bearing different letters are significantly different (*P* < 0.05).

It is well known that ROS is a by-product of aerobic metabolism and is involved in different signaling pathways and cell homeostasis. However, excessive ROS can induce apoptosis by damaging the DNA and other biomacromolecules (46). As shown in Figure 5, compared with the control group and the pre-treatment group, cells treated with H_2_O_2_ showed various changes, and the HEP-4 pre-treatment group showed significant inhibition of the production of ROS (Figure 5A). In addition, the protection of HepG2 cells by HEP-4 was found to be dose-dependent in the tested concentration range.

### Effects of HEP-4 on the activities of glutathione peroxidase catalase and catalase in H_2_O_2_-treated HepG2 cells

Glutathione peroxidase catalase and catalase are major antioxidant enzymes and play a crucial role in interception (47). Previous studies have demonstrated that plant polysaccharides can reduce cell apoptosis by increasing the activities of CAT and GSH-Px, thereby reducing H_2_O_2_-induced oxidative damage to hepatocytes (48). As expected, the results of this study showed that the activity of GSH-Px in H_2_O_2_-induced HepG2 cells was significantly increased after pre-treatment of HepG2 cells with different concentrations of HEP-4 (Figure 5B). Similarly, the CAT activity of HepG2 cells treated with H_2_O_2_ demonstrated a downward trend (Figure 5C), whereas, at a concentration of 800 μg/mL, the CAT activity of HepG2 cells pre-treated with HEP-4 was significantly increased, indicating that HEP-4 improved the inhibition of H_2_O_2_ on cell activity.

### Effects of HEP-4 on the content of malondialdehyde in H_2_O_2_-treated HepG2 cells

Following the H_2_O_2_-induced oxidative stress in HepG2 cells, the MDA levels modified significantly. For example, You et al. reported that polysaccharides from Panax notoginseng root extract inhibited H_2_O_2_-oxidative stress and reduced cell damage by decreasing ROS and MDA content (49). Consistent with this report, HEP-4 protected the cells from oxidative stress by eliminating ROS and reducing MDA levels. As shown in Figure 5D, the effect of HEP-4 on the MDA levels of HepG2 cells showed that the MDA levels of HepG2 cells were significantly increased after H_2_O_2_ treatment, whereas the MDA levels of HEP-4 group were lower than those of the H_2_O_2_ group (*P* < 0.05). When HEP-4 concentration was 800 μg/mL HEP-4, the MDA level of HepG2 cells decreased most significantly.

In summary, an oxidative damage cell model was established by H_2_O_2_ induction, and the cellular antioxidant capacity of honeysuckle polysaccharide HEP-4 was studied. The results showed that HEP-4 significantly reduced the levels of intracellular ROS and the degree of oxidative damage, whereas significantly increased the activities of antioxidant enzymes such as CAT and GSH-Px, and reduced the content of harmful substances such as MDA. Furthermore, a comprehensive analysis of structural characterization and *in vitro* antioxidant research results indicated that honeysuckle polysaccharide HEP-4 could be used as a novel natural antioxidant with potential application values in the food and pharmaceutical industries.

## Conclusion

In conclusion, a polysaccharide from honeysuckle was extracted by hot water, and the primary fraction HEP-4 was obtained by DEAE-52 and Sephadex G-75 columns, and its molecular weight was 1.98 × 10^5^ Da. The monosaccharide composition analysis results showed that HEP-4 was primarily composed of monosaccharides such as glucose, mannose, and galactose. In addition, a combination of periodate oxidation, Smith degradation, FI-IR, and NMR revealed that 1,4-β-D-Glc accounted for the majority of glycosidic bond types in polysaccharide components. HEP-4 exerts a strong antioxidant ability by scavenging ABTS⋅^+^ and DPPH⋅ free radicals. Furthermore, the results of the research showed that HEP-4 exerted a certain protective effect on HepG2 cells and protected them from oxidative stress damage. These results provide a basis for further understanding the polysaccharides obtained from honeysuckle and their applications in functional foods and pharmacology. However, the antioxidant effect and the structure–activity relationship of HEP-4 still require further comprehensive investigation to comprehensively elucidate the underlying mechanism. In addition, studies have reported that the application of modern chemical techniques, such as methylation, to chemically modify the structure of natural polysaccharides can improve their biological activity and even endow them with new biological activities, which is also the direction of our future work.

## Data availability statement

The original contributions presented in this study are included in the article/Supplementary material, further inquiries can be directed to the corresponding author.

## Author contributions

FA and GR: investigation, data curation, methodology, formal analysis, writing – original draft, and software. KC, ML, YML, and YFL: methodology, formal analysis, and writing – review and editing. XH and MS: data curation, validation, software, investigation, data curation, software, investigation. JW: supervision, funding acquisition, and project administration. RW: conceptualization, supervision, project administration, and writing – review and editing. All authors contributed to the article and approved the submitted version.
