# Supplementary material for: Extraction, purification, structural characterization, and antioxidant activity of a novel polysaccharide from Lonicera japonica Thunb
Source: Front Nutr. 2022 Nov 1;9:1035760. doi: 10.3389/fnut.2022.1035760 (PMC9664063; doi:10.3389/fnut.2022.1035760)
Supplement: Supplementary file 1 [file Table_1.DOC]

**Supplementary methods:**

**Supplementary method S1:**

Monosaccharide composition analysis

The sample (5 mg) were hydrolyzed in trifluoroacetic acid (2 M, 1 mL) at 100℃ for 6 h. Excess trifluoroacetic acid (TFA) was removed by rotating evaporator and the remaining tissue was dissolved in distilled water for PMP pre-column derivatization. On an Agilent Eclipse XDB-C18 column (5 μm, 4.6mm×250 mm) connected to the high performance liquid chromatography (HPLC) system, the derivatives were analyzed at 250 nm.

**Supplementary method S2:**

Methylation analysis

The sample (1 mg) was per-O-methylated with NaH-DMSO and methyl iodide. Afterward, the excess NaBH4 (1 M, 2 mL) was used for reduction and neutralised with 0.1 M acetic anhydride. Finally, the acetylated products were analyzed on a gas chromatography-mass spectrometry (GC-MS) system (Agilent 6890-5975C, USA) using an HP-5 column (30 m × 0.25 mm × 0.25µm).

**Supplementary method S3:**

DPPH radical scavenging assay

In brief, 0.5 mL of several solutions with five concentrations (0.2, 0.4, 0.6, 0.8, and 1.0 mg/mL) of polysaccharidewere mixed with DPPH solution (2.5 mL), respectively. The absorbance was measured at 517 nm after 30 min reaction at room temperature. In the same way, ethanol was used as a negative control, while Vc was used as a positive control at the same concentration. Each group was parallel for three times. The clearance rate of DPPH could be calculated by the following formula:

1.
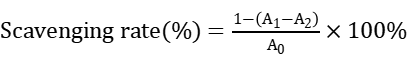


Where A_1_ is the absorption with DPPH solution and sample solution of different concentration; A_0_ is the absorption with DPPH solution and ethanol; A_2_ is the absorption with sample solution and ethanol.

**Supplementary method S4:**

Superoxide radical scavenging assay

With a minor modification 0.05 M Tris-HCI buffer solution (pH 8.2), 35 mM pyrogallol solution, and 8 mM HCl solution were prepared for use. 0.1mL of several solutions with different concentrations (0.2, 0.4, 0.6, 0.8, and 1.0 mg/mL) were mixed with 3mL of Tris-HCl buffer solution. After reacting for 8 min in a water bath at room temperature, 12 μL of pyrogallol was added to react for 5 min. Last, their absorbances at 320 nm were quickly measured respectively. Tris-HCl buffer solution was as a blank, and Ascorbic acid was as the control. The scavenging rate of superoxide anionwas calculated with the following equation:

1.
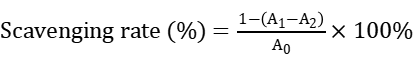


where A_0_ is the absorbance of distilled water instead of sample, A_1_ is the absorbance of the sample, A_2_ is the absorbance of distilled water instead of pyrogallol.

**Supplementary method S5:**

ABTS^•+^ radical scavenging assay

The ABTS^•+^ was generated by reacting 7 mM ABTS^•+^ solution of H_2_O and 2.45 mM potassium persulfate, and cultivated for 12 h in the dark at 25℃. Then the ABTS^•+^ solution was diluted with phosphate buffer solution (0.1 M, pH = 7.4) to adjust an absorbance of 0.701 ± 0.022 at 734 nm. 40 μL sample solution with different HEP concentrations (0.2, 0.4, 0.6, 0.8 and 1.0 mg/mL) was added into 0.2 mL ABTS^•+^ solution, the solution was then measured at 734 nm after incubation for 15 min at 20℃. Ascorbic acid was used as positive control set. The ABTS^•+^ scavenging activity was calculated according to the following formula:

1.
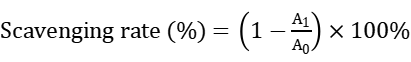


where A_0_ is the absorbance of the control sample, A_1_ is the absorbance of the tested sample.

**Supplementary method S6:**

Hydroxyl radical scavenging assay

The sample solution of 1 mL was put into plugged test tube, and 1 mL FeSO_4_ solution (9×10^-3^ mol/L), 1 mL salicylic acid-ethanol solution (9×10^-3^ mol/L) and 1 mL H_2_O_2_ solution (9×10^-3^ mol/L) were added. After reacted for 30 min at 37℃ with water bath, the ultraviolet absorbance of the mixture solution were measured at 510 nm respectively. Vc was used as the control. Each group was done for three times.

1.
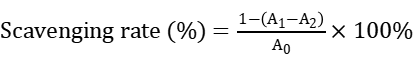


where A_0_ is the absorbance of absolute ethanol instead of sample, A_1_ is the absorbance of the sample, A_2_ is the absorbance of absolute ethanol instead of salicylic acid.

**Supplementary Figures**


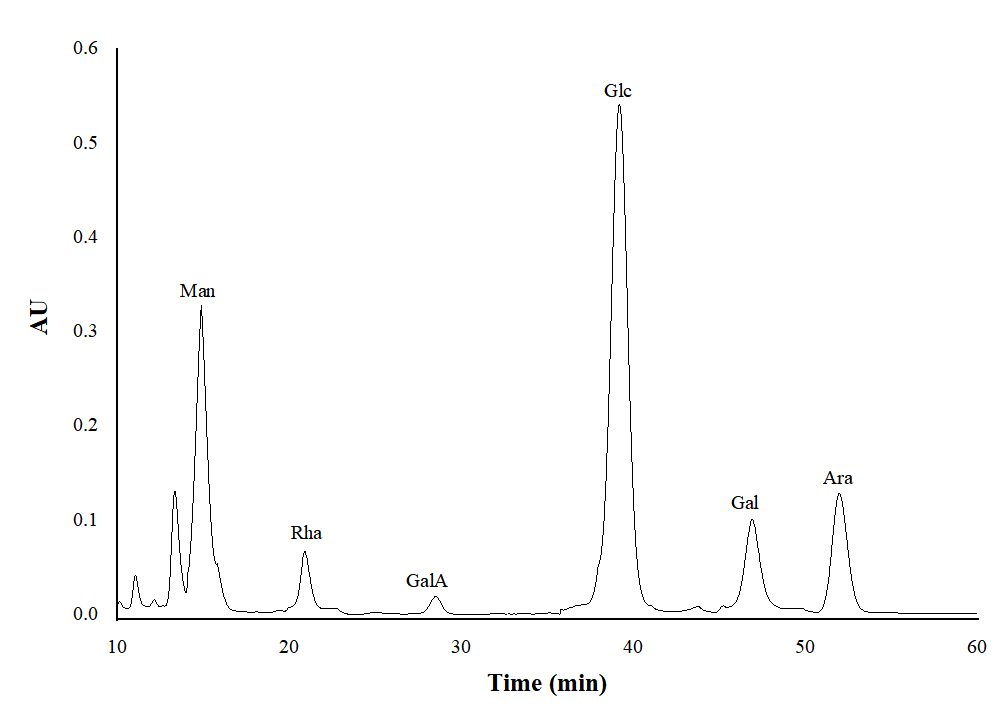


**Fig. S1.** Monosaccharide spectrum of HEP-4

**Fig. S2.**  Fourier-transform infrared spectra of HEP-4


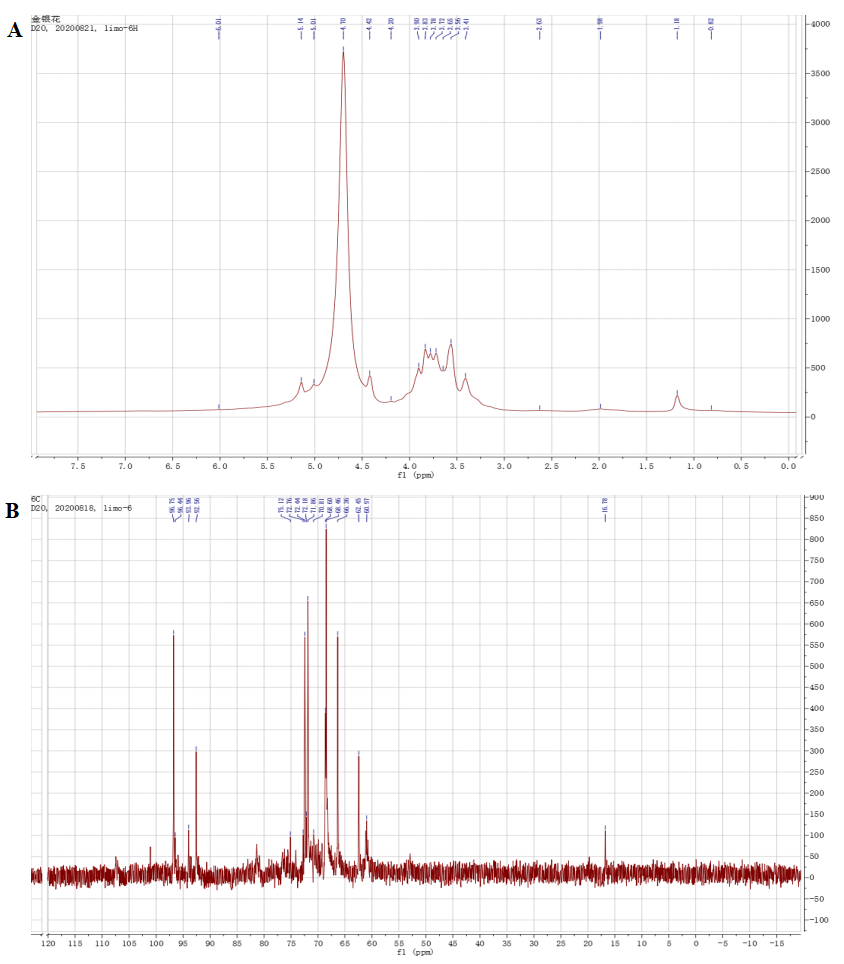


**Fig. S3.** The ^1^H-NMR spectrum (A) and ^13^C-NMR spectrum (B) of HEP-4
